# Online yoga to reduce post traumatic stress in women who have experienced stillbirth: a randomized control feasibility trial

**DOI:** 10.1186/s12906-020-02926-3

**Published:** 2020-06-05

**Authors:** Jennifer Huberty, Mariah Sullivan, Jeni Green, Jonathan Kurka, Jenn Leiferman, Katherine Gold, Joanne Cacciatore

**Affiliations:** 1 Arizona State University, Tempe, USA; 2 Colorado School of Public Health, Denver, USA; 3 University of Michigan Medical School, Ann Arbor, USA

**Keywords:** Infant death, Mental health, Mindfulness, wWomen’s health

## Abstract

**Background:**

About 1 in every 150 pregnancies end in stillbirth. Consequences include symptoms of post traumatic stress disorder (PTSD), depression, and anxiety. Yoga has been used to treat PTSD in other populations and may improve health outcomes for stillbirth mothers. The purpose of this study was to determine: (a) feasibility of a 12-week home-based, online yoga intervention with varying doses; (b) acceptability of a “stretch and tone” control group; and (c) preliminary efficacy of the intervention on reducing symptoms of PTSD, anxiety, depression, perinatal grief, self-compassion, emotional regulation, mindfulness, sleep quality, and subjective health.

**Methods:**

Participants (*N* = 90) were recruited nationally and randomized into one of three groups for yoga or exercise (low dose (LD), 60 min per week; moderate dose (MD), 150 min per week; and stretch-and-tone control group (STC)). Baseline and post-intervention surveys measured main outcomes (listed above). Frequency analyses were used to determine feasibility. Repeated measures ANCOVA were used to determine preliminary efficacy. Multiple regression analyses were used to determine a dose-response relationship between minutes of yoga and each outcome variable.

**Results:**

Over half of participants completed the intervention (*n* = 48/90). Benchmarks (≥70% reported > 75% satisfaction) were met in each group for satisfaction and enjoyment. Participants meeting benchmarks (completing > 90% of prescribed minutes 9/12 weeks) for LD and MD groups were 44% (*n* = 8/18) and 6% (*n* = 1/16), respectively. LD and MD groups averaged 44.0 and 77.3 min per week of yoga, respectively. The MD group reported that 150 prescribed minutes per week of yoga was too much. There were significant decreases in PTSD and depression, and improvements in self-rated health at post-intervention for both intervention groups. There was a significant difference in depression scores (*p* = .036) and grief intensity (*p* = .009) between the MD and STC groups. PTSD showed non-significant decreases of 43% and 56% at post-intervention in LD and MD groups, respectively (22% decrease in control).

**Conclusions:**

This was the first study to determine the feasibility and preliminary efficacy of an online yoga intervention for women after stillbirth. Future research warrants a randomized controlled trial.

**Trial registration:**

ClinicalTrials.gov. NCT02925481. Registered 10–04-16.

## Background

Every year 2.6 million babies are stillborn worldwide, and one in 150 babies die before or during birth in the United States (U.S.) alone [1]. Stillbirth is defined in the U.S. as the death of a baby from 20-weeks gestation to birth [2, 3]. Stillbirth is unique because it involves birth and death in a single, often very traumatic, event. Bereaved mothers are seven times more likely to to have symptoms of post-traumatic stress disorder (PTSD), four times more likely to be diagnosed with clinical depression, and twice as likely to be diagnosed with an anxiety disorder [4]. Symptoms of PTSD have been reported to last anywhere from 2 months to 18 years [5]. The long-lasting adverse effects of stillbirth may even impact the health of the mother and child in subsequent pregnancies.

Women often become pregnant relatively quickly after a stillbirth (50–98% of pregnancies within 12–18 months) and yet may not be emotionally or physically ready for another child [6, 7]. This may lead to dysregulation of family life leading to psychological and other problems in subsequently born children [8]. Mothers who have experienced stillbirth are more likely to report symptoms of anxiety (22.5%) and depression (19.7%) during subsequent pregnancies compared to women whose babies are born alive [9]. In addition, poor and emotional health outcomes have been associated with adverse health outcomes (i.e., poor self-rated health) which can affect physical well-being in subsequent pregnancies [5]. Thus, there is a need for inter-conception care (time between stillbirth and the subsequent pregnancy or decision not to conceive again) for bereaved mothers after the stillbirth of a baby.

There is currently no standard of care during the inter-conception period after stillbirth. A recent systematic review of experimental interventions revealed only two interventions to improve emotional, mental, or physical health after a stillbirth in the last 36 years [10]. In one study, families met with a grief support team twice within 6 months and there were no differences in grief at post intervention compared to the control [11]. The second study was a mindfulness-based feasibility study for anxiety and depression with two sessions over a two-week time period but only six participants completed both sessions, limiting the generalizability of the findings [12]. Mothers often report seeking help through support groups, psychotherapy, and medication [13] to help cope with the stressors of traumatic grief. Considering the negative health implications following stillbirth and risks of poor mental, emotional, or physical health in subsequent pregnancies, interventions are desperately needed.

Yoga may be a feasible intervention to improve women’s well-being during the inter-conception period after stillbirth. Yoga is a non-phamacologic option for women who want to avoid medication in the inter-conception period due to concern for the subsequent baby and possible adverse effects. Yoga brings together the practice of breathing, physical postures, and meditation [14] and has been utilized for decades to reduce stress and disease [15]. It has been reported to reduce symptoms of PTSD, anxiety, and depressive symptoms in a number of populations [15, 16], and it is a complementary approach to reduce stress during pregnancy and aiding in postpartum depression [17]. In a small non-randomized trial in mothers after stillbirth, Huberty and colleagues reported the majority of participants were satisfied with a 60 min per week online yoga intervention with reported benefits such as reducing mental and physical stress, improved mindfulness and calm, and getting more time for themselves [18].

While the exact mechanism through which yoga reduces symptoms of PTSD, anxiety, and depression is still unknown, changes may be attributable, at least in part, to improvements in mindfulness, emotional regulation, self-compassion, and/or sleep quality. For example, bereaved mothers often report recurring flashbacks, hypervigilance, hyperarousal, rumination, and avoidance [19]. Through the cultivation of mindfulness, yoga provides the practitioner with a means to focus attention and increase awareness of the mind and body. Enhanced mindfulness may increase acceptance of one’s own emotions, improving regulation and reducing avoidance [20]. Yoga may also bring a deeper awareness of negative beliefs about oneself (such as self-criticism) [20] and promote self-compassion [21–24].

Yoga may also improve sleep quality. A reduction in ruminating thoughts, hypervigilance, and hyperarousal may lower cortisol levels in the body, promoting parasympathetic nervous system activity [22, 25, 26]. This improves both the ability to fall asleep and stay asleep. This is important as studies suggest dysregulated sleep patterns may predict PTSD symptoms [27]. Yoga may improve comorbid conditions associated with PTSD including depression and anxiety, giving it high utility as an adjunctive treatment to cope with grief after stillbirth, especially during the inter-conception period [28].

Yoga has the potential to improve mental, emotional, and physical health in these bereaved mothers, hence, the purpose of this study was to determine the feasibility (i.e., acceptability, demand) of a 12-week, home-based, online-streamed yoga intervention with varying doses (low = 60 min per week; moderate = 150 min per week). We also explored the acceptability of an active control group to be used in a future efficacy trial. Finally, we determined the preliminary effects (not powered for efficacy) of the intervention on reducing symptoms of PTSD, anxiety, depression, and perinatal grief and the proposed mechanism of self-compassion, emotional regulation, mindfulness, sleep quality, and self-rated health.

## Methods

### Study design

This was a three-group randomized feasibility trial. The Institutional Review Board at Arizona State University approved this study. All participants provided their consent to participate and granted the research team permission to collect medical records before beginning the study. This study adheres to CONSORT guidelines.

#### Participant recruitment and selection

Participants were recruited using snowball sampling through stillbirth-related non-profit organizations (e.g., Stories of Babies Born Still, Star Legacy Foundation, Mothers in Sympathy and Support Foundation), social media (e.g., Facebook, Instagram), and hospital/clinics. Research staff asked organizations to advertise the study by posting recruitment information (e.g., fliers, memes) in clinics or to their social media sites, websites, newsletters, and listservs. Participants could receive up to $65 for completing each component of the intervention (e.g., questionnaires, interviews). Recruitment occurred from July 2017 until September 2018, and the intervention occurred from August 2017 until May 2018. The intervention concluded when all participants (*N* = 90, 30 per group) had completed the intervention. As this was a feasibility study, the sample was appropriate for early phase-development trials and consistent with previously conducted beta tests that demonstrated a 20% attrition rate [29]. Relatively small sample sizes are common in feasibility studies [29], thus a post-hoc sample size analysis was conducted based on observed effect size of the primary outcome PTSD.

Interested participants were directed to a web-based eligibility screener via Qualtrics. See Table 1 for eligibility criteria. Though pregnant women were excluded from the study, if a woman became pregnant during the study she could still participate if she completed a PARmed-X for Pregnancy (a screening tool for prenatal exercise) and received approval from her physician. Ineligible participants were notified by phone or email and were offered a discounted membership to Udaya, the yoga platform used for this study. Eligible participants were sent an email to schedule an intake phone call to explain study procedures, the informed consent, and the HIPAA authorization form. After the intake phone call, participants were sent a link to sign an informed consent document and complete a baseline questionnaire. The research team faxed a copy of the signed informed consent and HIPAA authorization form to the physician’s office to release the participants’ medical records to the research team.

**Table 1:** Inclusion and exclusion criteria

Inclusion	Exclusion
Experienced a stillbirth within the past 6 weeks to 24 months	Unstable psychiatric condition (i.e., psychosis; suicidal ideation with plan)
Clinical levels of posttraumatic stress symptoms (score of ≥33 on the Impact of Events Scale)	Pregnant at time of enrollment
≥18 years of age	Practiced yoga at least 60 min/week
Resided in the U.S.	Scored 20–27 on the Patient Health Questionnaire-9 (i.e., severe depression)
Able to read/understand/speak English	At risk for suicide based on follow-up phone assessment after positive screen (score of 1, 2, or 3 on the last question on the PHQ-9)
Underactive (≤120 min/week of moderate intensity physical activity)	
Willing to be randomized	
Regular internet access via mobile phone, desktop/laptop computer, tablet etc.	
Answered “no” to all items on the PAR-Q (i.e., can participate in exercise safely) or given clearance by a doctor	

#### Randomization

Following baseline assessments, women were enrolled into one of the following three groups for 12 weeks: 1) intervention low dose (LD) = 60 min/week yoga (*n* = 30), 2) intervention moderate dose (MD) = 150 min/week yoga (*n* = 30), or 3) stretch and tone control group (STC) = 60 min/week of stretching/toning exercises (*n* = 30). A list of randomized participant numbers were generated using randomizer.org by a colleague who was not a member of the research team. The list was concealed from members of the research team and revealed only when it had been assigned to a participant. Members of the research team would then enroll participants based on their assigned numbers.

#### Procedures

Once participants were assigned to a group, the research team mailed the intervention group a package containing study information and directions, one yoga mat, two blocks (i.e., brick-shaped prop to assist in reaching the floor), and one yoga strap (i.e., long cloth to help increase range of motion). Participants in the control group were also sent a package with study information and directions, a stretching mat, and one resistance band. Participants were asked to notify the research team once they received their package. Once the research team was notified, participants were provided an on-line username and password to begin the intervention via email.

#### Intervention

The intervention groups (LD and MD) were asked to follow a 12-week online yoga prescription developed by the research team [18]. Briefly, the prescription included 12 videos developed for women who had experienced stillbirth [18] and the remaining 48 videos were chosen using Udaya’s existing library of more than 600 yoga videos. Udaya is an online streaming platform that provides yoga videos that vary in length, style, and language, instructed by experienced yoga teachers (e.g., 200 h or more of yoga training). All videos were appropriate for women up to 20 weeks gestation. Udaya yoga videos were Hatha based, which combines a physical practice with a meditative component to cultivate mindfulness (i.e., moment-to-moment, non-judgmental awareness, cultivated by paying attention in specific ways) [30] and self-awareness. Hatha yoga includes instruction from a variety of lineages (e.g., Iyengar, Ashtanga, Vinyasa). Both the LD and MD groups had identical poses and sequences in each prescription. Participants were asked to complete the yoga videos in a specific order, which included specialized preparatory instructions for each video to ensure comfort and safety.

#### Control

Participants in the stretch and tone control (STC) group were asked to follow a 12-week online stretching/toning exercise prescription for 60 min per week to match the LD intervention group. The research team developed 12, 30-min videos for the STC group prescription (produced and filmed by Udaya). These videos were developed by adapting a well-established evidence-based protocol specific for women who are underactive [30]. Each video included a three-minute warm up and cool down, and a stretch or tone exercise for 1–2 sets of 20–45 s or 10–15 repetitions.

### Measures

All data were collected online via Qualtrics.

#### Demographics

Demographic data collected at baseline included date of birth, race/ethnicity, household income, education level, marital status, occupation, height and weight. Participants were asked if they had a history of chronic health conditions (e.g., hypertension), history of post-traumatic stress disorder and/or clinical depression, were were currently on medications for mental health, and for a rating of their overall physical health. Finally, participants were asked whether or not they were currently participating in any in-person and/or online support groups.

#### Feasibility

All feasibility measures were assessed at post-intervention (12-weeks). We measured feasibility using Bowen’s guidelines [31] including acceptability, demand, and preliminary efficacy (i.e., *trends* in changes on reducing symptoms of PTSD, anxiety, depression, and perinatal grief and the proposed mechanism of self-compassion, emotional regulation, mindfulness, sleep quality, and self-rated health.

##### Acceptability

We assessed acceptability by ability to perform/comply with the intervention and satisfaction. Ability to perform/comply was measured using number of dropouts and number of participants who completed each part of the intervention (i.e., daily and weekly logs, self-reported outcomes, and satisfaction survey). Participants were asked to self-report their online yoga/exercise sessions in an online daily log including which video they completed, date, and start/end times. Satisfaction was measured with an investigator-developed survey of 29 questions (e.g., Likert-scale, yes/no, options). Benchmarks for satisfaction related to enjoyment, intervention, and instruction/instructors were that at least 70% of participants would report 75% satisfaction (see Table 2).

**Table 2:** Satisfaction

Question	Response	*n*
Enjoyment		
Overall, how much did you enjoy your online classes?	Low dose group	
	Very much	11 (61)
	Somewhat	6 (33)
	Not at all	1 (6)
	Moderate Dose	
	Very much	7 (50)
	Somewhat	7 (50)
	Not at all	-
	Control	
	Very much	5 (36)
	Somewhat	9 (64)
	Not at all	-
Did you enjoy doing exercise/yoga at home?	Low Dose	
	Yes	17 (94)
	No	1 (6)
	Moderate Dose	
	Yes	14 (100)
	No	-
	Control	
	Yes	13 (93)
	No	1 (7)
Prescription		
Were you able to complete your prescribed amount (i.e., 60 or 150 minutes) of online videos each week?	Low Dose	
	Yes	12 (67)
	No	6 (33)
	Moderate Dose	
	Yes	3 (20)
	No	12 (80)
	Control	
	Yes	7 (50)
	No	7 (50)
I felt the prescribed minutes (i.e., 60 or 150 minutes) per week of online videos was:	Low Dose	
	Too much	1 (6)
	About right	15 (88)
	Too little	1 (6)
	Moderate Dose	
	Too much	8 (57)
	About right	6 (43)
	Too little	-
	Control	
	Too much	-
	About right	10 (71)
	Too little	4 (29)
Satisfaction		
I am satisfied with participating in the online streaming videos	Low Dose	
	Strongly agree	7 (39)
	Agree	8 (44)
	Neutral	2 (11)
	Disagree	1 (6)
	Moderate Dose	
	Strongly agree	8 (57)
	Agree	5 (36)
	Neutral	-
	Disagree	1 (7)
	Control	
	Strongly agree	9 (64)
	Agree	5 (36)
	Neutral	-
	Disagree	-
I am satisfied with the instructor(s) in the online videos	Low Dose	
	Strongly agree	7 (39)
	Agree	6 (33)
	Neutral	4 (22)
	Disagree	1 (6)
	Moderate Dose	
	Strongly agree	9 (64)
	Agree	5 (36)
	Neutral	-
	Disagree	-
	Control	
	Strongly agree	10 (71)
	Agree	4 (29)
	Neutral	-
	Disagree	-
Coping		
The online streaming videos has helped me to cope with the grief associated with the death of my baby	Low Dose	
	Strongly agree	5 (28)
	Agree	7 (39)
	Neutral	4 (22)
	Disagree	2 (11)
	Moderate Dose	
	Strongly agree	5 (36)
	Agree	5 (36)
	Neutral	3 (21)
	Disagree	1 (7)
	Control	
	Strongly agree	3 (21)
	Agree	5 (36)
	Neutral	4 (29)
	Disagree	2 (14)
Easiness		
The classes were easy	Low Dose	
	Strongly agree	4 (19)
	Agree	5 (28)
	Neutral	6 (33)
	Disagree	3 (17)
	Moderate Dose	
	Strongly agree	-
	Agree	6 (43)
	Neutral	6 (43)
	Disagree	2 (14)
	Control	
	Strongly agree	-
	Agree	8 (57)
	Neutral	4 (29)
	Disagree	2 (14)
The instruction in the videos was easy to follow	Low Dose	
	Strongly agree	6 (33)
	Agree	9 (50)
	Neutral	2 (11)
	Disagree	1 (6)
	Moderate Dose	
	Strongly agree	4 (29)
	Agree	9 (64)
	Neutral	1 (7)
	Disagree	-
	Control	
	Strongly agree	11 (79)
	Agree	3 (21)
	Neutral	-
	Disagree	-
It was easy to find the time to do the videos	Low Dose	
	Strongly agree	2 (11)
	Agree	8 (44)
	Neutral	4 (22)
	Disagree	3 (17)
	Strongly disagree	1 (6)
	Moderate Dose	
	Strongly agree	-
	Agree	3 (21)
	Neutral	3 (21)
	Disagree	6 (43)
	Strongly disagree	2 (14)
	Control	
	Strongly agree	1 (7)
	Agree	5 (36)
	Neutral	4 (29)
	Disagree	3 (21)
	Strongly disagree	1 (7)
Continue/Recommend		
I will continue participating in online streaming exercise or yoga in the future	Low Dose	
	Strongly agree	2 (11)
	Agree	11 (61)
	Neutral	4 (22)
	Disagree	-
	Strongly disagree	1 (6)
	Moderate Dose	
	Strongly agree	7 (50)
	Agree	2 (14)
	Neutral	3 (21)
	Disagree	-
	Strongly disagree	2 (14)
	Control	
	Strongly agree	4 (29)
	Agree	7 (50)
	Neutral	2 (14)
	Disagree	1 (7)
	Strongly disagree	-
I will recommend participating in online streaming exercise or yoga to other women who have experienced stillbirth	Low Dose	
	Strongly agree	8 (44)
	Agree	9 (50)
	Neutral	1 (6)
	Disagree	-
	Moderate Dose	
	Strongly agree	9 (64)
	Agree	2 (14)
	Neutral	2 (14)
	Disagree	1 (7)
	Control	
	Strongly agree	8 (57)
	Agree	5 (36)
	Neutral	-
	Disagree	1 (7)
Social Support		
If we added an online social support group, how would it have affected your participation?	Low Dose	
	Would have participated in MORE videos	5 (50)
	It wouldn't have changed anyting	2 (20)
	I don't know	3 (30)
	Would have participated in LESS videos	-
	Moderate Dose	
	Would have participated in MORE videos	6 (86)
	It wouldn't have changed anyting	1 (14)
	I don't know	-
	Would have participated in LESS videos	-
	Control	
	Would have participated in MORE videos	5 (71)
	It wouldn't have changed anyting	-
	I don't know	2 (29)
	Would have participated in LESS videos	-
If we were to conduct a future study, do you think we should add an online social support group (i.e., Facebook group, discussion board)?	Low Dose	
	Yes	11 (100)
	No	-
	Moderate Dose	
	Yes	7 (88)
	No	1 (12)
	Control	
	Yes	7 (100)
	No	-

##### Demand

Demand was determined by participants reporting they would recommend yoga to others, continue to participate, and that the program was easy to complete. Benchmarks were that at least 70% of participants would report they agree to each construct, measured with the satisfaction survey. Demand was also determined using attendance to the intervention; analytics tracking from the Udaya website via Wistia plugin, a software that tracks engagement with online videos [29]. Data collected included title of the video, date and time it was completed, number of minutes per session, total number of minutes completed each week. Number of yoga activity minutes per week were compiled primarily from self-report logs while supplementing the Wistia plugin values when self-report logs were missing. Benchmarks for the intervention groups were set for participants to complete at least 90% of the respective group’s prescribed weekly dose of yoga minutes (LD = 55 min; MD = 135 min) on at least 9 out of the 12 weeks (75%).

### Preliminary efficacy

All preliminary efficacy measures were collected at baseline, post-intervention (12-weeks), and follow-up (20-weeks).

#### Impact of event scale

Post-traumatic stress disorder symptoms were measured using the Impact of Event Scale (IES-R). The IES-R was developed to reflect the criteria for PTSD per the Diagnostic Symptom Manual (DSM-IV-TR). The scale consists of 22 questions which are scored on a five-point Likert scale (0 = not at all, 1 = a little bit, 2 = moderately, 3 = quite a bit, 4 = extremely). There are three subscales (i.e., avoidance, intrusion, hyperarousal) and the sum of the three subscales scores comprise the total score. Other studies have reported subscale scores to have high internal consistency (α = 0.79–0.92). A total score ≥ 33 indicates the “best cutoff for a probable diagnosis for PTSD”. Possible scores range from 0 to 88 [32].

#### State-trait anxiety inventory

The State-Trait Anxiety Inventory (STAI) yields scores indicating levels of trait (Form Y-1) and state (Form Y-2) anxiety, with higher scores indicating greater levels of anxiety. This scale is scored using a four-point Likert scale (1 = not at all, 2 = somewhat, 3 = moderately so, 4 = very much so), and participants respond to questions such as, “I feel calm,” and “I feel nervous and restless.” The STAI has demonstrated reliability in pregnant and postpartum populations. Possible scores range from 20 to 80 [33].

#### Patient health Questionnaire-9

The Patient Health Questionnaire-9 (PHQ-9) was used to measure depressive symptoms. This measure is commonly used to screen, diagnose, monitor, and measure the severity of depression. Scores range from 0 to 27 and cut-off scores of 5, 10, 15, and 20 indicate mild, moderate, moderately severe, and severe depressive symptoms, respectively. This scale is valid and reliable in general populations (α = .86–.89) and has been validated in pregnant and postpartum populations [34, 35].

#### Perinatal grief scale

The Perinatal Grief Scale (PGS) was used to measure symptoms of grief after perinatal loss. It is a valid 33-item instrument with three subscales (i.e., active grief, difficulty coping, and despair) with higher scores indicating more intense perinatal grief. Possible scores range from 33 to 165 [36].

#### Self-compassion scale

The Self-Compassion Scale (SCS) is a 26-item questionnaire using a five-point Likert scale from 1 = almost never to 5 = almost always, The SCS consists of six subscales (self-kindness, self-judgment, common humanity, isolation, mindfulness, over-identified) and has good construct validity and reliability (α = .92). A total score is calculated by taking the mean of each subscale and reverse scoring the negative subscale items (i.e., self-judgment, isolation, over-identification) and computing a total mean. Higher scores indicate higher levels of self-compassion and possible scores range from 1 to 5. A sample question includes, “When times are really difficult, I tend to be tough on myself,” [37].

#### Emotion regulation questionnaire

The Emotion Regulation Questionnaire (ERQ) is a 10-item scale used to measure an individual’s tendency to regulate his or her emotions by two strategies: cognitive reappraisal and expressive suppression. Most alphas exceed .80. Higher scores indicate greater use of emotional regulation strategies [38, 39].

#### Mindful attention awareness scale

The Mindful Attention Awareness Scale (MAAS) is a 15-item scale that measures the extent to which individuals are able to maintain awareness of present-moment experience. This scale uses a 6-point Likert scale (ranging from 1 = almost always to 6 = almost never), and the mean is computed to generate a total score. Higher scores indicate higher levels of mindfulness and possible scores range from 1 to 6. This scale is a valid and reliable measure with good internal consistency (α = .80–.87) [40].

#### Pittsburgh sleep quality index

Sleep was measured subjectively using the Pittsburgh Sleep Quality Index (PSQI). The PSQI is a 19-item questionnaire that has demonstrated reliability and validity in pregnant populations [41]. Higher global PSQI scores indicate more sleep disturbances and possible scores range from 0 to 21.

*SF-12*. The SF-12 is a measure of self-reported health developed as a shorter version (12-item) of the original SF-36 Health Survey. The physical health composite score was used in this study. Scores range from 0 to 100, with zero being the lowest level of health and 100 the highest [42].

### Data analysis

Descriptive analyses were performed for demographic characteristics using means and standard deviation of continuous data and frequencies and proportions of discrete data for each intervention group. One-way analysis of variance were conducted to examine between-group differences on demographic characteristics. Frequency analyses were used to determine feasibility (acceptability and demand), compliance, and outcome measures using only participants that completed both baseline and post intervention surveys. To test the preliminary efficacy of the trial, repeated measures (collected at baseline, post-intervention, follow-up time points) analysis of covariance analyses (ANCOVA) were performed for each of the outcome measures of PTSD, anxiety (state and trait), depression, perinatal grief, self-compassion, emotional regulation and suppression, mindfulness attention awareness, self-rated health, and subjective sleep quality (PSQI). Lastly, to investigate the dose of yoga activity related to changes in the outcome measures, multiple regression analyses were conducted using the pooled sample (i.e. no group stratification) for each outcome for all participants with baseline and post-intervention data.

ANCOVA analyses were conducted independently on each outcome while controlling for age, race, household income, education level, marital status, BMI, and the level of peer support received (none, online, in-person, both online and in-person). Regression models were built using a hierarchical approach, adding demographic variables based on theoretical importance while consindering multicollinearity indices. All analyses were conducted while applying appropriate adjustments (i.e. Greenhouse-Gesser corrections when sphericity was not met, Bonferroni corrections for post hoc analyses) to account for non-parametric data and multiple comparisons and effect sizes were computed [43] using SPSS [44] with a statistical significance threshold set at *p* < .05. Post-hoc analysis to determine required sample size to achieve statistical significance (α < 0.05) with appropriate power (1- β = 0.80) for repeated measures within-between interaction effects was conducted using G*Power 3.1.9.4.

## Results

### Participants

**Fig. 1:**
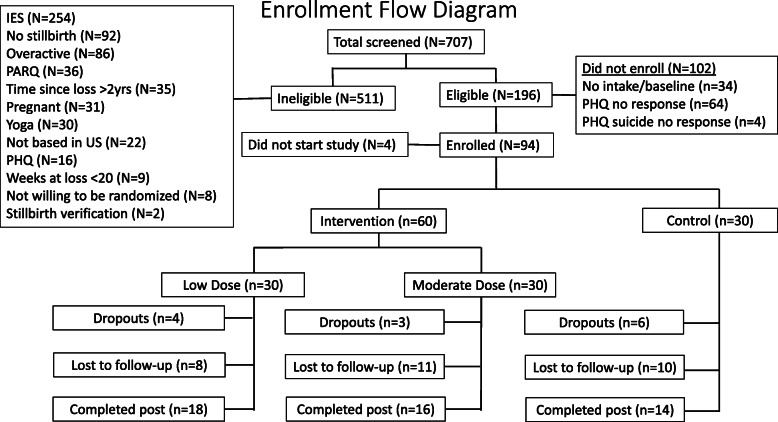
CONSORT diagram

A total of 707 participants completed the eligibility questionnaire. Of those, 196 participants met the eligibility criteria and 94 enrolled in the study. Four enrollees did not start the study, yielding a total of 90 participants. Once eligibile but prior to randomization, 30% of women (*n* = 30) were asked to verify their stillbirth (i.e., doctor’s note) with 28 verified and two unverified and ineligible. Sixty participants were randomized to the intervention groups (LD *n* = 30; MD *n* = 30), and 30 participants were randomized to the control group (see Fig. 1).

### Demographics

Participants were predominantly White (*n* = 78; 86%). Most participants (*n* = 53; 59%) earned $61,000 per year or more, were college educated (baccalaureate *n* = 32; 36% and graduate *n* = 20; 22%), and married (*n* = 75; 83%). The average gestational age at the time of stillbirth was 30 weeks (SD = 6.99). See Table 3 for further demographic information.

**Table 3:** Participant demographics

	n	%
Race & Ethnicity		
Hispanic	9	10
Non-Hispanic	80	88.9
American Indian or Alaskan Native	1	1.1
Asian or Asian-American	1	1.1
Native Hawaiian or Pacific Islander	1	1.1
White, European-American, Caucasian	78	86.7
Black, African American, Nativ African	5	5.6
Bi-racial or Multi-racial	3	3.3
Other	1	1.1
Income		
Less than $20,000	8	8.9
$21,000-40,000	16	17.8
$41,000-60,000	12	13.3
$61,000 and above	53	58.9
Education		
High school diploma	5	5.6
Some college	20	22.2
Associates	12	13.3
Bachelors degree	32	35.6
Graduate degree or above	20	22.2
Martital Status		
Single	6	6.7
Partnered/In a relationship	5	5.6
Married	75	83.3
Divorced	3	3.3
	Mean	SD
Stillbirth		
Weeks gestiation at time of loss	30.43	6.99
Time since stillbirth	40.92	29.17
	n	%
*Delivery method*		
Vaginal	79	87.8
Cesarean Section	11	12.2
*Know cause of death?*		
Yes	47	52.2
No	43	47.8
*Did baby survive at all?*		
Yes	3	3.3
No	87	96.7

### Feasibility

#### Acceptability

##### Ability to perform/comply with intervention

Thirteen participants (14%) formally dropped out of the study (LD *n* = 4, MD *n* = 3, STC *n* = 5) and 11 completed a survey about the reasons for withdrawal. Twenty-nine participants did not complete the study (i.e., did not formally drop out, but did not continue to participate in yoga and did not complete the post intervention surveys) (See Fig. 1). Reasons for drop out varied and included mood, pregnancy, time, and stress. Participants who dropped out reported they enjoyed the online streaming videos (*n* = 7; 63.6%) and thought an online social support group would have helped them continue participating in the study (*n* = 7; 55%). More than one-half of participants completed the post-intervention and satisfaction survey (*n* = 48/90; LD *n* = 18; MD *n* = 16; STC *n* = 14) (See Table 4).

**Table 4:** Ability to comply with intervention components

	Low Dose	Moderate Dose	Control
Daily logs	201/360 (56%)	160/360 (44%)	148/360 (41%)
Weekly logs	201/360 (56%)	160/360 (44%)	148/360 (41%)
Baseline	90/90 (100%)	90/90 (100%)	90/90 (100%)
Post	18/30 (60%)	16/30 (53%)	14/30 (46%)
Dropout	4	3	5
Lost to follow up	8	11	10

##### Satisfaction

Benchmarks (≥70% would report 75% satisfaction) were met for all groups related to satisfaction of the intervention including enjoyment and instruction/instructors. Benchmarks (> 70%) for prescribed minutes being appropriate were met in the LD and STC groups but not in the MD group (See Table 2).

Nearly all participants (*n* = 45; 98%) reported that online yoga affected their lives in a positive way due to increased calmness, relaxation, enhanced ability to handle life and process emotions, and more time for self, though reported benefits varied by group (i.e., intervention groups v control). Almost 70% of participants reported they would have participated more if the study included a support group and 96% reported social support should be included in future studies (See Table 2). Barriers to participation in the study (*n* = 25/48; 52%) included work/school commitments, care for others, mood, and time. No adverse injuries were reported during this study but there were some reports of discomfort (*n* = 8, 12%), though these included normal symptoms of exercise (e.g., muscle soreness, wrist/ knee discomfort). Seven participants became pregnant during the study (LD *n* = 4, MD *n* = 1, STC n = 2) and five of those reported pregnancy was a barrier to participation in the study for reasons including being afraid of hurting the baby and fatigue. Half of all participants who completed the intervention reported experiencing a technical limitation while watching the videos (LD *n* = 7, MD *n* = 6, STC *n* = 10). Across all groups, the most commonly reported technical limitation was bad or slow internet connection (*n* = 15).

#### Demand

Approximately 60% of participants would recommend online yoga to others who have experienced stillbirth, and that they would continue to participate in online yoga and thought the program was easy to complete. Benchmarks for demand (≥70% in all categories), however, were not met in any group (See Table 2). Benchmarks for attendance (i.e., time spent in yoga; completing at least 90% of the prescribed minutes at least 9/12 weeks) were not met in any of the groups. The percent of participants meeting benchmarks for LD and MD groups were 44% (*n* = 8/18) and 6% (n = 1/16), respectively. Across the intervention, LD and MD groups averaged 44 and 77 min per week of yoga, respectively. Participants over-reported online yoga participation compared to Wistia analytics tracking across the 12-week study.

#### Preliminary efficacy

**Fig. 2:**
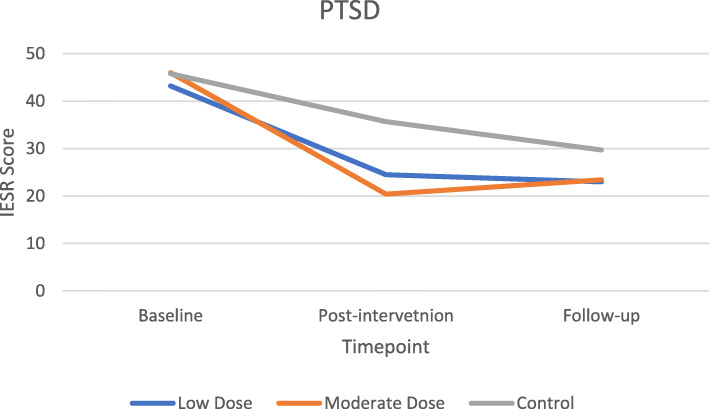
Change in PTSD scores by timepoint

**Fig. 3:**
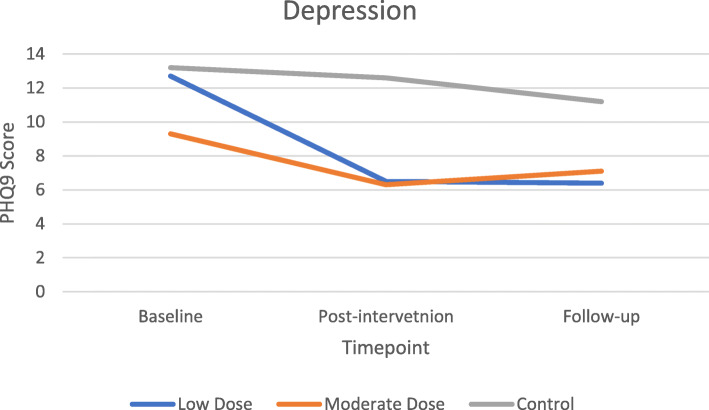
Change in depression by timepoint

**Fig. 4:**
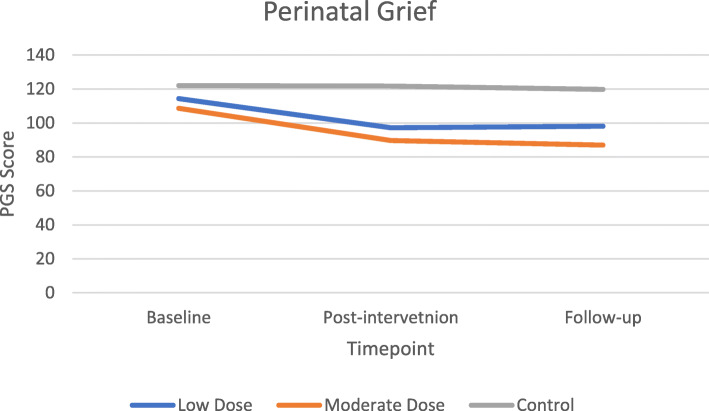
Change in perinatal grief by timepoint

There were no significant differences in demographics or peer-support between groups at the baseline time point. The primary outcome of IES-R had no main effect from intervention group (*F* (2, 22) = 1.460, *p* = .254, η^2^ = 0.117), but did decrease throughout the trial (*F*(1.511, 33.237) = 5.231, *p* = 0.017, η^2^ = 0.192), specifically from baseline to post-intervention (Fig. 2). There was no significant interaction between IES-R and intervention group, indicating that regardless of intervention group, PTSD was not affected by group assignment. Secondary outcomes of depression (*F* (2, 21) = 3.90, *p* = 0.036, η^2^ = 0.271) and perinatal grief *F* (2, 22) = 5.830, *p* = 0.009, η^2^ = 0.346) showed significant effect between intervention groups with expected differences in LD and MD groups compared to the STC. Depression (*F*(1.486, 31.20) = 6.788, *p* = 0.007, η^2^ = 0.244) and self-compassion (*F* (2, 46) = 3.229, *p* = 0.049, η^2^ = 0.123) showed improvements over the course of the trial (Figs. 3 & 4). Self-rated health (*F*(1.580, 34.749) = 3.830, *p* = 0.041, η^2^ = 0.148) decreased over the course of the trial in the LD and STC groups. Nonsignificant differences between groups indicated MD blunted this self-perceived diminution *F*(3.159, 34.749) = 0.592, *p* = 0.633, η^2^ = 0.051).

Although no interactions were found for any outcome between groups over time with effect sizes ranging from 0.01–0.156, the primary outcome of PTSD showed non-significant decreases of 43 and 56% (the largest relative change among outcome measures) at post-intervention for the LD and MD groups, respectively, compared to a 22% decrease in the STC (*F* (2, 22) = 2.212, *p* = 0.133, partial η^2^ = 0.167). Post-hoc sample size analysis for the intervention group-by-repeated IES-R measure interaction effect size (η2 = 0.105) determined that 171 participants (57 per group) would be required to achieve statistical significance. Sleep quality also showed non-significant improvements of 34% in the LD group and 29% in the MD group compared to a 10% worsening in the STC between baseline and post-intervention (*F* (2, 21) = 3.148, *p* = 0.064, partial η^2^ = 0.231). Finally, perinatal grief remained static in the STC throughout the trial while MD and LD groups showed non-significant decreases from baseline to post-intervention (*F* (2, 22) = 1.769, *p* = 0.194, η^2^ = 0.139) that was maintained at follow-up. Outcomes of anxiety (STAI), self-compassion (SCS), emotional regulation (ERQ), and mindfulness (MAAS) showed visual improvements in the hypothesized direction but no significant differences. See Table 5 for mean differences by timepoint.

**Table 5:** Mean differences of intervention x timepoint

		Means (95% CI)		
Outcome	Time point	Low Dose	Moderate Dose	Study Control
Impact of Events (PTSD)				
	pre-intervention	43.16 (35.77–50.55)	45.99 (39.30–52.68)	45.75 (36.81–54.69)
	post-intervention	24.53 (16.72–32.34)	20.38 (13.31–27.45)	35.67 (26.22–45.12)
	follow-up	23.03 (13.86–32.20)	23.39 (15.08–31.69)	29.67 (18.57–40.77)
Anxiety (STAI)				
	pre-intervention	91.38 (82.78–99.98)	86.68 (79.36–94.01)	90.67 (80.80–100.55)
	post-intervention	87.80 (80.72–94.89)	86.06 (80.03–92.09)	88.77 (80.63–96.90)
	follow-up	88.17 (81.60–94.75)	82.36 (76.76–87.95)	85.19 (77.65–92.74)
Depression (PHQ)				
	pre-intervention	12.67 (9.23–16.11)	9.27 (6.28–12.26)	13.15 (9.14–17.17)
	post-intervention	6.46 (3.50–9.42)	6.31 (3.74–8.89)	12.56 (9.10–16.02)
	follow-up	6.38 (3.57–9.19)	7.11 (4.66–9.56)	11.21 (7.92–14.49)
Self-compassion (SCS)				
	pre-intervention	2.52 (2.06–2.98)	2.79 (2.31–3.28)	2.46 (1.85–3.07)
	post-intervention	2.88 (2.40–3.37)	2.86 (2.35–3.37)	2.61 (1.97–3.26)
	follow-up	2.83 (2.34–3.32)	2.85 (2.33–3.37)	2.54 (1.89–3.20)
Emotional Regulation (ERQ)				
	pre-intervention	17.63 (14.42–20.84)	19.42 (16.38–22.46)	22.62 (18.73–26.51)
	post-intervention	17.75 (14.37–21.12)	16.89 (13.69–20.09)	20.20 (16.10–24.29)
	follow-up	16.10 (11.91–20.30)	19.06 (15.09–23.03)	18.29 (13.20–23.37)
Self-rated Health (SF12)				
	pre-intervention	61.19 (56.79–65.60)	57.03 (53.04–61.02)	59.05 (53.72–64.38)
	post-intervention	57.26 (52.94–61.57)	57.24 (53.33–61.15)	58.04 (52.81–63.26)
	follow-up	56.08 (51.32–60.84)	57.14 (52.83–61.45)	55.93 (50.17–61.69)
Perinatal Grief (PGS)				
	pre-intervention	114.42 (103.51–125.34)	108.70 (98.82–118.59)	122.11 (108.91–135.31)
	post-intervention	97.21 (84.31–110.12)	89.70 (78.01–101.38)	121.77 (106.15–137.38)
	follow-up	98.12 (81.05–115.19)	86.88 (71.42–102.35)	119.73 (99.07–140.39)
Sleep Quality (PSQI)				
	pre-intervention	11.40 (9.13–13.67)	8.87 (6.72–11.01)	8.26 (5.51–11.01)
	post-intervention	7.52 (5.25–9.78)	6.26 (4.11–8.40)	9.10 (6.35–11.84)
	follow-up	7.42 (5.15–9.68)	5.60 (3.46–7.75)	6.82 (4.08–9.57)

#### Dose response

To determine the dose response of yoga activity on PTSD compared to STC, hierarchical regression was conducted in which predictors were added based on theoretical importance while checking for multicollinearity. A series of models were built, first controlling for depression, BMI, age, household income, education level, race, and peer support. Marital status was not included due to lack of variability in STC. Due to multicollinearity determined from significant correlations (*p* < 0.05) with BMI, household income (*r* = −.41, *p* = 0.009), education level (*r* = −.41, *p* = 0.009), and race (*r* = .36, *p* = 0.021) were not included in the final model, which controlled for BMI, age, and peer support. Participating in yoga was a non-significant predictor of reduced IES-R scores (*t* (32) = − 0.50, β = − 0.031, 95% CI [− 0.196, 0.095], *p* = 0.621). contradictory to participating in the STC group *t* (12) = 0.121, β = 0.046, 95% CI [− 0.924, 1.016], *p* = 0.908). For participants in the LD or MD groups, 10-min increases in yoga activity weekly for 12 weeks (i.e. 120 min increase averaged over 12 weeks) resulted in a 3.72 unit decrease in PTSD whereas the STC resulted in a 5.52 unit increase in PTSD.

## Discussion

The purpose of this study was to determine the feasibility and acceptability of a 12-week, home-based, online-streamed yoga intervention with varying doses (low = 60 min/week; moderate = 150 min/week) among bereaved mothers who had experienced the death of a baby to stillbirth. This was the first randomized controlled study to determine the feasibility of online yoga in grieving mothers. Although just under half the participants did not finish the study, many of those who dropped out mentioned that they enjoyed the online videos (almost 64%) and thought social support online would have helped them complete the study (55%). Overall participants were satisfied with the intervention and thought it affected them in a positive way. Only a small number of participants met the benchmarks for demand, yet there were significant reductions in depression and perinatal grief in the MD group as compared to the STC. Additionally, there were notable non-significant reductions in PTSD symptoms and improvements in sleep quality. With both intervention groups showing similar trends for improvement, these preliminary efficacy data also suggest that lower doses of yoga may be enough to elicit reductions in post traumatic stress symptoms.

### Acceptability

Just under 46% of participants did not finish the study (i.e., dropped out or did not complete post), mainly because of time, mood (e.g., lack of energy and motivation), and stress. It was not surprising that time was a major reason for not completing the study as other physical activity interventions have also revealed time as a barrier, especially in pregnant and post-partum populations and women in the inter-conception period [45, 46]. These studies report that childcare and family obligations consume the time that they might have for physical activity/exercise [46]. Women also reported mood as a reason for not completing the study. Participants in our study were within 2 years of experiencing a baby’s death. Some literature suggests symptoms related to post traumatic stress are most prevalent and severe in the first year following the loss, and these symptoms interfere with normal life activities [19]. Mood-related symptoms associated with PTSD may have inhibited motivation to complete the prescribed videos each week. Although the prevalence and patterns of mood-related symptoms after stillbirth are not known, one study found that women diagnosed with PTSD after live-birth had higher levels of anxiety, depression, and parenting stress through 12 months postpartum [47]. The time since the baby’s death could play a key role in the ability of a woman to participate in inter-conception care (e.g., delivery of intervention based on time since the stillbirth) [48], and this information could inform the design of future interventions. Additionally, individuals experiencing symptoms of post-traumatic stress may perceive everyday stressors as more severe than prior to the traumatic event and may not have proper coping mechanisms to handle minor stressors [49]. If the intervention was perceived as an added stressor (e.g., another item on a to-do list), participants may have not been motivated to participate. That is, they may be more inclined to avoid these stressors until they have developed coping mechanisms to manage stressors.

Interestingly, participants who dropped out of the study reported they enjoyed participating in online yoga or exercise and thought if the study included an online social support group they would have been more likely to continue. Social support is a well-known behavior strategy to increasing compliance and attendance to behavioral interventions [50] Social support is critical for women after stillbirth as many report feeling isolated and their grief is often disenfranchised [51]. It is a key component in coping with loss and trauma, and those who report lower levels of social support have reported more adverse health outcomes [52] Social support may also play a role in the effects of yoga on mental health outcomes as it has been reported that social support provided during classes may be a coping mechanism for stress [53] Because the kind of support that would naturally occur in an in-person class was not present in this online intervention, there is a need to determine feasible, alternative support strategies.

### Satisfaction

Overall, participants were satisfied with the intervention, reporting they enjoyed the intervention and instructors. Similar to those who dropped out of the intervention, most participants reported they would have participated more if there had been an online social support group. Social support has been used in various physical activity interventions in order to increase adherence and enjoyment, and in some populations has been thought to mediate the effects of the intervention [54].

In the participants who became pregnant during the study and completed the intervention, many reported discomfort and being afraid of hurting the baby as barriers to participation. These women also did not have the opportunity to receive advice for modifications as they would have in an in-person prenatal yoga class. In order to progress the feasibility of online yoga interventions, future studies should consider including an online mode of delivery for a yoga instructor to provide feedback and assistance in order to prevent injury and offer modifications. For example, participants could use a real-time video software to engage with an instructor while actually performing the poses. Yoga instructors may offer support and a feeling of safety, ensure proper techniques are employed, and increase motivation.

Another obstacle with online yoga is the potential for technical issues. Most participants who reported experiencing technical issues said it was due to slow internet connections. One way to address this in future studies might be to provide instruction and recommendations for the participants to download the videos before watching them to complete their sessions.

When asked what barriers kept them from participating in yoga (on a weekly basis), women reported similar barriers to the women that dropped out of the study (i.e., work, caring for others, mood, time). In our study, we provided a barriers workbook (i.e., tips for overcoming barriers to participation) and weekly reminders to participate. The literature is not conclusive regarding overcoming barriers to exercise, but reminders seem to be effective for some populations [55]. Because of the high dropout rate and number of women who reported barriers, there is a need to develop strategies for fidelity to an online yoga intervention. We utilized reminders to participate in yoga or STC but did not include any behavioral strategies or language to overcome barriers within these reminders. Future studies could include reminders with tips that are specific to women who have had a stillbirth (i.e., sensitivity to grief, awareness of other responsibilities such as children). Additionally, because social support is necessary for improving health outcomes after a stillbirth, future research may utilize social support as a means to overcome barriers. For instance, women in the study could connect with other women who are participating and discuss ideas for overcoming their barriers such as scheduling time and childcare, and provide emotional support for each other.

### Demand

Most women in the MD group reported 150 min per week to be too much and, not surprisingly, did not meet the benchmarks. Interestingly, although most participants in the low dose group reported a dose of 60 min per week of yoga to be appropriate, less than half of participants met the benchmarks for participation. Low fidelity is not surprising considering the high distress within this population [56] Other studies in populations with PTSD symptoms have reported similar compliance rates [57] Schottenbauer and colleagues found that it was common for dropout rates to be around 50% in high posttraumatic stress populations [58]. Reinhardt and colleagues also reported low retention rates in a yoga intervention for veterans with PTSD [57]. In a yoga intervention for survivors of domestic violence, Clark and colleagues reported a 30% dropout rate [59]. More research is needed to determine intervention strategies to increase fidelity specific to this population.

### Preliminary efficacy

Because most participants did not meet the benchmarks for minutes of yoga participation per week, preliminary efficacy results are interpreted with average minutes of actual participation per week (LD = 44, MD = 77) rather than prescribed minutes per week (LD = 60, MD = 150). There was a significant reduction in posttraumatic stress and depressive symptoms at post-intervention but not at follow-up. Other studies have shown short-term effects of yoga on depressive and PTSD symptoms (e.g., 20) but more research is needed to determine the long-term effects of yoga on these outcomes. The physical domain of self-rated health decreased over the course of the intervention. Our findings are inconsistent to self-rated health outcomes in yoga interventions overall [60]. However, women who have experienced a stillbirth report physical health declines and may be more likely to develop chronic illness [61]. Although women in the STC and LD groups experienced decreases in their physical self-rated health, women in the MD group did not have any changes. This suggests yoga may have mitigated the typical decline in self-rated health seen in mothers who experience stillbirth. More research is needed in this population, specifically, to determine implications of self-rated health as it relates to post traumatic stress and other negative psychological and physical health outcomes.

The significant decreases in depression and perinatal grief in the MD group compared to the STC group suggests that 77 min of yoga per week may be enough to improve these outcomes. Other studies have shown similar improvements in depressive symptoms with 60–75 min of yoga per week compared to control groups (e.g., [62, 63]) It should be noted that most yoga interventions have been conducted using in-person formats as opposed to the online, home-based format in the current study. Therefore, discrepancies between number and length of sessions are inherent despite the potentially identical number of minutes per week. More research is needed to determine the differences and efficacy between the two methods. Additionally, although yoga may decrease depressive symptoms and grief, both states may also be barriers to participation and less likely to participate [64].

Although not significant, trends in improvements of post traumatic stress symptoms, perinatal grief, and sleep quality in both LD and MD groups, compared to the STC group, also suggest the intervention may contribute to these changes regardless of dose, a concept further supported when additionally considering regression results. Decreases on the IES-R in both intervention groups was the largest relative change among all outcome measures, suggesting a potential for yoga to decrease symptoms at doses of both 44 and 77 min.

Our dose-response findings, while also non-significant, suggested potential decreases in post traumatic stress and depression scores for every 10-min increase of yoga per week. Taken together, these trends suggest low doses of yoga (approximately 20 min on average) may be enough to elicit positive changes but more minutes per week may yield greater magnitudes of improvement. That said, these findings should also be considered in light of the feasibility findings previously discussed. That is, even though there may be an increase in benefits with additional participation, participants reported 60 min to be feasible whereas 150 min was too much. More research is needed to determine the point at which benefits are balanced with feasibility.

### Limitations

This was the first study to determine the feasibility of online yoga in women who have experienced stillbirth and therefore had limitations, many related to the online nature of the intervention. First, there were inconsistencies with the software used to track participation (i.e., viewing the videos) and data for some participants was not accurately recorded. Therefore, the research team utilized self-report data and the results were subject to biases that are inevitable with this type of data (e.g., social desirability). In the satisfaction surveys, participants reported technical issues that limited viewing the videos (e.g., slow internet) and may have inhibited their participation and the ability to track the minutes appropriately. Future studies should consider ways to overcome such issues that are likely inherent with online interventions. Second, the sample was not necessarily representative of the population of the women who have had stillbirths in the US, as most were white and higher income. Although in a previous study we asked minority women who had experienced stillbirth about cultural barriers to recruitment and about their perceptions and opinions on yoga [18], additional recruiting methods are needed for representative samples to ensure generalizability of the results. Third, this study had a high number of dropouts. Participants had within the last 2 years, experienced a traumatic event, and may be coping with a number of stressors. Future studies should consider strategies recommended above (i.e., social support) to increase retention and adherence to an online yoga intervention. The nature of social media recruitment may have led to a self-selection bias in this study. Finally, this was a feasibility study and thus our sample size was not powered to determine efficacy. That is, conclusions cannot be made about the effect of yoga on any of the preliminary outcome measures (e.g., PTSD, anxiety, depression). Though findings showed trends in the hypothesized directions, future studies warrant sample sizes powered to determine efficacy.

## Conclusion

Online yoga may be an effective and feasible strategy to reduce depressive symptoms, perinatal grief, and symptoms of PTSD in mothers who have experienced stillbirth. More research is needed to determine strategies to increase compliance, the most appropriate number of minutes per week in order to experience changes in outcomes, and the effects of the intervention compared to a control group.

## Data Availability

Available from the corresponding author upon reasonable request.

## Abbreviations

PTSD: Post-traumatic stress disorder
LD: Low dose intervention group
MD: Moderate dose intervention group
STC: Stretch and tone control group
IES-R: Impact of Event Scale-Revised
STAI: State-Trait Anxiety Inventory
PHQ-9: Patient Health Questionnaire-9
PGS: Perinatal Grief Scale
SCS: Self-Compassion Scale
ERQ: Emotion Regulation Questionnaire
MAAS: Mindful Attention Awareness Scale
PSQI: Pittsburgh Sleep Quality Index
SF-12: Self-Rated Health Survey-Short Form
